# Large Intra-Mural Myoma Removed by Enucleation

**Published:** 1880-11

**Authors:** 


LARGE INTRA-MURAL MYOMA REMOVED BY ENUCLEATION. (Under the care of Mr. LAWSON TAIT.)

A. H., aged twenty-eight, was admitted into the hospital on May 15th, suffering from profuse menorrhagia. The fundus was found to be retroflected to an extreme degree, and of very large size. The diagnosis of myoma was made, and an attempt to dilate the cervix by Mr. Tait's plugs was made, but it was unsuccessful owing to the extreme retroflexion. The cervix was therefore completely divided by the hysterotome, the thick capsule of the tumor opened, and the tumor separated as far as the fingers could reach. The patient was so extremely anæmic that everything done to her was done in the constant fear of the production of heart-clot, and even the administration of ether in her case seemed risky. Nearly eight weeks elapsed after this preliminary proceeding before it was deemed safe to do anything more, when it was found that the opening into the capsule had closed, though the division of the cervix had remained permanent. On July 26th the dilating plugs were applied, and next day the cervix was so open that a large free incision over the tumor was made, and the tumor freely separated. On August 4th the tumor was found presenting through the os, and a large piece of it was removed by the écraseur. On August 12th the rest of it was found extruded and hanging by a thick pedicle, which was divided and the mass removed. For many weeks after this operation she hung between life and death, and made a convalescence protracted over nearly eight months.

Mr. Tait is of opinion that in such a case removal of the ovaries is a much safer operation than enucleation of the tumor, and in his hands it has given much better results. Out of five cases where he has performed vaginal enucleation in the last three years, there have been only two recoveries, whilst of seven similar cases in which he has performed oöphorectomy there has been only one death, and that death was due solely to the operation having been too long delayed.

